# Perceptions of large language models in medical education and clinical practice among pediatric emergency physicians in Saudi Arabia: a multiregional cross-sectional study

**DOI:** 10.3389/fpubh.2025.1634638

**Published:** 2025-07-30

**Authors:** Yara AlGoraini, Mashhour Alsayyali, Ola Alotaibi, Ibtihal Almeshawi, Fahad Alaifan, Rawan Alrashed

**Affiliations:** ^1^Pediatric Emergency Department, King Fahad Medical City, Riyadh, Saudi Arabia; ^2^Pediatric Emergency Department, Armed Forced Hospital Alhada, Taif, Saudi Arabia

**Keywords:** artificial intelligence, ChatGPT, pediatric emergency medicine, medical education, clinical decision support, physician perceptions, Saudi Arabia, digital health

## Abstract

**Background:**

Artificial intelligence (AI) is reshaping healthcare delivery and education, but little is known about its perceived value among pediatric emergency medicine (PEM) physicians in Saudi Arabia. This study aimed to assess the perceptions and experiences of PEM physicians in Saudi Arabia toward the use of AI, particularly ChatGPT, in clinical practice and medical education.

**Methods:**

A cross-sectional, web-based survey was conducted among 100 PEM physicians across various regions of Saudi Arabia. The questionnaire explored demographics, AI experience, perceived benefits and limitations, and the evaluation of ChatGPT-generated clinical and educational content.

**Results:**

Most participants (96%) believed that AI tools, such as ChatGPT, would play a significant role in the future of PEM. A high agreement was observed regarding AI’s usefulness in medical education (91%) and clinical practice, particularly in differential diagnosis (77%) and documentation (78%). The ChatGPT-generated responses to a clinical scenario (croup) were rated highly for validity, reasoning, and educational value. However, 66% of them still preferred traditional textbooks for complex topics. The key concerns included accuracy (83%), patient safety (56%), and lack of regulatory guidance (52%).

**Conclusion:**

Saudi PEM physicians show a strong interest in integrating AI tools, such as ChatGPT, into clinical and educational workflows. Although optimism is high, concerns about safety, ethics, and oversight highlight the need for regulatory frameworks and structured implementation strategies.

## Background

Artificial intelligence (AI) is increasingly recognized as a transformative force in health care that offers novel solutions for clinical decision-making, workflow optimization, and medical education. One prominent tool, ChatGPT, which is a generative AI model developed by OpenAI, has sparked significant interest because of its ability to simulate clinical reasoning, generate patient-specific educational content, and support evidence-based practices. In recent years, AI tools, such as ChatGPT, have gained attraction in various medical settings because of their potential to enhance efficiency and reduce the cognitive load for clinicians (1).

In pediatric emergency medicine (PEM), where time-sensitive decisions and continuous education are critical, AI is a promising adjunct. Globally, AI can aid in clinical documentation, differential diagnosis, and real-time guideline adherence (2–6). In education, AI-powered platforms are increasingly used to deliver customized learning experiences, simulate clinical scenarios, and provide rapid access to vast medical knowledge (4, 5).

The selection of Pediatric Emergency Medicine (PEM) was intentional due to the high cognitive demands, rapid clinical decision-making, and continuous educational requirements inherent in this field. These characteristics make PEM particularly well-positioned to benefit from AI support, yet it remains underexplored compared to other high-acuity specialties such as cardiology and neurology.

Despite these advances, the integration of AI in health care widely varies across regions, with developing countries facing unique challenges, including limited digital infrastructure, regulatory uncertainty, and cultural considerations (7). In Saudi Arabia, recent national initiatives under Vision 2030 have accelerated the digital health transformation, positioning AI as a key enabler of medical innovation (8). However, empirical data on the perception and adoption of LLMs, such as ChatGPT, among frontline physicians—particularly in emergency pediatrics—remain scarce. While global studies have begun exploring ChatGPT’s educational and clinical potential, few have examined clinician perspectives in non-Western or resource-variable settings. This study aims to fill that gap by capturing physician views on ChatGPT’s utility in PEM, thus informing culturally relevant implementation strategies and contributing to the broader discourse on responsible LLM integration.

## Methods

### Study design

This cross-sectional, questionnaire-based study assessed the perceptions and experiences of PEM physicians in Saudi Arabia regarding the use of AI, particularly ChatGPT, in clinical practice and medical education.

### Study population and sample size

Participants included board-certified consultants, fellows, and specialists practicing PEM across various regions of Saudi Arabia. Participants were selected from the five major geographical regions of Saudi Arabia (central, eastern, western, northern, and southern). This multiregional sampling aimed to reflect regional variability in clinical workflows, digital infrastructure, and exposure to AI-based tools. The estimated number of pediatric emergency physicians working in Saudi Arabia is 500 (200 consultants + 100 assistant consultants + 200 fellows). Assuming that 10% of the pediatric emergency physicians responded to the online survey, a margin of error of 15% on both sides of the response rate was established, and the finite population was applied considering the total population, which determined an optimal sample size of *n* = 55 for the proposed study.

### Data collection tool

A structured, web-based questionnaire was specifically developed for this study to explore perceptions of AI in pediatric emergency medicine. It was designed and developed by an expert input to ensure content relevance. The questionnaire was reviewed by a panel of five experts in pediatric emergency medicine and medical education to assess content validity (Supplementary Appendix A). A pilot test was conducted with 10 physicians to evaluate clarity and flow, resulting in minor revisions. The final tool demonstrated good internal consistency (Cronbach’s alpha > 0.80) and adequate face and content validity. The survey was structured into four sections to comprehensively assess the perceptions and experiences of pediatric emergency physicians toward AI use. The first section collected demographic and professional data, including age, sex, region, training level, and years of experience. The second section used a 5-point Likert scale to evaluate general perceptions of and experiences with AI tools, such as ChatGPT, in clinical and educational settings. The third section involved a standardized ChatGPT-generated response to a fictional clinical scenario involving a 3-year-old child with moderate croup symptoms. The prompt used was: *“How would you manage a 3-year-old child presenting to the ED with moderate croup?”* This was entered into ChatGPT 4o (OpenAI) in April 2024, and the output was reviewed by five PEM experts for accuracy, completeness, and clinical relevance.

The complete questionnaire, including the clinical case and ChatGPT response, is provided in Supplementary Appendix A. The final section included open-ended questions that allowed participants to share their opinions on the benefits, limitations, and ethical concerns of AI in health care.

To ensure the quality of the tool, the survey was developed based on existing literature and reviewed by a panel of five experts in PEM and medical education for content validity. A pilot test was conducted with 10 physicians to assess clarity and flow, resulting in minor revisions. Internal consistency for the Likert scale items was confirmed, with Cronbach’s alpha > 0.80, indicating good reliability. The final survey demonstrated adequate face and content validity for the intended population.

The full questionnaire, including the croup scenario and the ChatGPT-generated response, is available as Supplementary Appendix A.

### Data analysis

Data were analyzed using the Statistical Package for Social Science version 20. Descriptive statistics were used to summarize participants’ demographics and response patterns. Categorical variables are expressed as frequencies and percentages. Continuous variables are reported as means with standard deviations or medians with interquartile ranges, as appropriate. Chi-squared and independent t-tests were used to compare AI users and nonusers. Logistic regression analysis was performed to identify the factors associated with AI adoption. A *p*-value < 0.05 was considered statistically significant.

### Ethical considerations

Ethical approval was obtained from the Institutional Review Board of King Fahad Medical City (IRB00010471). Informed consent was obtained from all participants prior to data collection.

## Results

### Demographic characteristics

In total, 100 participants were included in this study (Table 1). Most participants (65.0%) were between age 20–39 years, 34.0% were between 40 and 59 years, and only 1.0% were 60 ≥ years. The sample consisted predominantly of males (62.0%) rather than females (38.0%). Most participants were of Saudi nationality (77.0%), with non-Saudi participants accounting for 23.0% of the sample. Regarding marital status, 58.0% of the participants were married, 38.0% were single, 3.0% were divorced, and 1.0% were separated. In terms of professional level, consultants represented the largest group (45.0%), followed by fellows (38.0%), and assistants/specialists (17.0%). The educational background of the participants varied, with 46.0% holding doctorate degrees or board certifications, 44.0% having completed fellowship training, 9.0% with master’s degrees, and 1.0% with bachelor’s degrees.

**Table 1:** Sociodemographic characteristics of PEM physicians (*n* = 100).

Variables		Total population, *n* = 100 (%)
Age	20–39 years	65 (65.0)
	40–59	34 (34.0)
	60+	1 (1.0)
Sex	Female	38 (38.0)
	Male	62 (62.0)
Nationality	Non-Saudi	23 (23.0)
	Saudi	77 (77.0)
Marital status	Divorced	3 (3.0)
	Married	58 (58.0)
	Separated	1 (1.0)
	Single	38 (38.0)
Professional level	Assistants/specialists	17 (17.0)
	Consultant	45 (45.0)
	Fellow	38 (38.0)
Educational level	Bachelor’s degree	1 (1.0)
	Doctorate or board-certified	46 (46.0)
	Fellowship	44 (44.0)
	Master’s degree	9 (9.0)
Residence	Central region	47 (47.0)
	Eastern region	19 (19.0)
	Northern region	7 (7.0)
	Southern region	8 (8.0)
	Western region	19 (19.0)
Employment	Employed	71 (71.0)
	Trainee	28 (28.0)
	Unemployed	1 (1.0)
Do you believe AI tools, such as ChatGPT, will play a major role in the future of PEM?	Yes	96 (96.0)
	No	4 (4)
Total years of clinical experience in pediatric emergency medicine	Min–max	1–17
	Mean ± SD	9 ± 4.7
	Median (P25–P75)	9 (5–13)

The participants were distributed across different regions of Saudi Arabia, with the highest representation from the central region (47.0%), followed by equal representation from the eastern and western regions (19.0% each), the southern region (8.0%), and the northern region (7.0%). Most participants were employed (71.0%), whereas 28.0% were trainees, and 1.0% were unemployed.

Notably, an overwhelming majority of participants (96.0%) believed that AI tools, such as ChatGPT, would play a major role in the future of PEM, with only 4.0% disagreeing with this statement. The clinical experience of the participants in PEM ranged from 1 to 17 years, with a mean of 9 years (standard deviation = 4.7) and a median of 9 (interquartile range, 5–13) years.

### Perceptions toward artificial intelligence use in medical education

The participants demonstrated highly positive perceptions toward the use of AI in medical education (Table 2). Most respondents (91.0%) agreed or strongly agreed that AI, such as ChatGPT, could enhance medical education by providing quick access to medical knowledge (mean score = 4.38 ± 0.65). Similarly, 66.0% agreed or strongly agreed that AI tools, such as ChatGPT, improved decision-making and critical thinking skills in trainees (mean score = 3.84 ± 0.88).

**Table 2:** Participants’ perception toward AI use in medical education and clinical practice (*n* = 100).

Statements	Strongly disagree (%)	Disagree (%)	Neutral (%)	Agree (%)	Strongly agree (%)	Mean ± SD	Total
Perception toward AI use in medical education							
AI, such as ChatGPT, can enhance medical education by providing quick access to medical knowledge	0 (0.0)	0 (0.0)	9 (9.0)	44 (44.0)	47 (47.0)	4.38 ± 0.65	100 (100%)
AI tools, such as ChatGPT, improve decision-making and critical thinking skills in trainees	0 (0.0)	7 (7.0)	27 (27.0)	41 (41.0)	25 (25.0)	3.84 ± 0.88	
AI tools, such as ChatGPT, can be effectively used to simulate patient cases for clinical learning	1 (1.0)	1 (1.0)	26 (26.0)	40 (40.0)	32 (32.0)	4.01 ± 0.85	
AI tools, such as ChatGPT, can supplement traditional teaching methods in PEM training	0 (0.0)	5 (5.0)	14 (14.0)	49 (49.0)	32 (32.0)	4.08 ± 0.81	
AI tools, such as ChatGPT, help in staying updated with the latest medical guidelines and literature	1 (1.0)	5 (5.0)	19 (19.0)	40 (40.0)	35 (35.0)	4.03 ± 0.92	
I believe AI-powered tools, such as ChatGPT, should be integrated into medical education curricula	1 (1.0)	5 (5.0)	19 (19.0)	35 (35.0)	40 (40.0)	4.08 ± 0.94	
Perception toward AI use in clinical practice							
ChatGPT medical responses are generally accurate	0 (0.0)	12 (12.0)	28 (28.0)	45 (45.0)	15 (15.0)	3.63 ± 0.88	100 (100%)
I am comfortable using AI-based tools, such as ChatGPT, for clinical decision support	1 (1.0)	18 (18.0)	22 (22.0)	42 (42.0)	17 (17.0)	3.56 ± 1.01	
AI tools, such as ChatGPT, can assist in differential diagnosis and treatment recommendations in PEM	0 (0.0)	3 (3.0)	20 (20.0)	50 (50.0)	27 (27.0)	4.01 ± 0.77	
AI tools, such as ChatGPT, can improve efficiency in documentation and chart reviews	0 (0.0)	0 (0.0)	22 (22.0)	50 (50.0)	28 (28.0)	4.06 ± 0.71	
AI-driven medical chatbots should be integrated into PEM workflows	1 (1.0)	3 (3.0)	22 (22.0)	44 (44.0)	30 (30.0)	3.99 ± 0.86	
I would trust AI-generated recommendations if validated by evidence-based medicine	1 (1.0)	4 (4.0)	18 (18.0)	47 (47.0)	30 (30.0)	4.01 ± 0.86	

Regarding clinical learning, 72.0% of the participants agreed or strongly agreed that AI tools, such as ChatGPT, could be effectively used to simulate patient cases (mean score = 4.01 ± 0.85). A substantial majority (81.0%) agreed or strongly agreed that AI tools, such as ChatGPT, could supplement traditional teaching methods in PEM training (mean score = 4.08 ± 0.81).

Most participants (75.0%) agreed or strongly agreed that AI tools, such as ChatGPT, helped in staying updated with the latest medical guidelines and literature (mean score = 4.03 ± 0.92). Furthermore, 75.0% of the respondents agreed or strongly agreed that AI-powered tools, such as ChatGPT, should be integrated into medical education curricula (mean score = 4.08 ± 0.94).

### Perceptions toward AI use in clinical practice

Regarding clinical practice applications, 60.0% of the participants agreed or strongly agreed that ChatGPT medical responses were generally accurate (mean score = 3.63 ± 0.88). A majority (59.0%) reported feeling comfortable using AI-based tools, such as ChatGPT, for clinical decision support (mean score = 3.56 ± 1.01). A substantial proportion (77.0%) agreed or strongly agreed that AI tools, such as ChatGPT, could assist in differential diagnosis and treatment recommendations in PEM (mean score = 4.01 ± 0.77). Similarly, 78.0% agreed or strongly agreed that AI tools, such as ChatGPT, could improve efficiency in documentation and chart reviews (mean score = 4.06 ± 0.71).

Most participants (74.0%) agreed or strongly agreed that AI-driven medical chatbots should be integrated into PEM workflows (mean score = 3.99 ± 0.86). A similar proportion (77.0%) indicated they would trust AI-generated recommendations if validated by evidence-based medicine (mean score = 4.01 ± 0.86).

### Evaluation of ChatGPT’s clinical response on croup

Participants rated the ChatGPT-generated response for croup highly in terms of clinical reasoning, validity, and usefulness in both patient care and education. For example, 85% of participants rated the response as having high validity, and 88% found it clinically reasonable (Table 3).

**Table 3:** Participants’ perception toward ChatGPT-generated responses about croup scenario in medical education, evidence-based medicine, and clinical practice (*n* = 100).

Statements	Strongly disagree (%)	Disagree (%)	Neutral (%)	Agree (%)	Strongly agree (%)	Mean ± SD	Total
ChatGPT-generated responses about clinical practice in croup disease							
The validity of this ChatGPT-generated response is high	0 (0.0)	3 (3.0)	12 (12.0)	51 (51.0)	34 (34.0)	4.16 ± 0.75	100 (100%)
The clinical reasoning demonstrated in this ChatGPT-generated response is strong	0 (0.0)	2 (2.0)	10 (10.0)	58 (58.0)	30 (30.0)	4.16 ± 0.68	
This ChatGPT-generated response is useful for clinical application	0 (0.0)	0 (0.0)	19 (19.0)	44 (44.0)	37 (37.0)	4.18 ± 0.73	
This ChatGPT-generated response is useful for patient education	0 (0.0)	0 (0.0)	12 (12.0)	50 (50.0)	38 (38.0)	4.26 ± 0.66	
I believe I can provide a better response than the ChatGPT-generated response for a given clinical scenario	2 (2.0)	5 (5.0)	26 (26.0)	44 (44.0)	23 (23.0)	3.81 ± 0.92	
This AI-generated response from ChatGPT could be beneficial for medical education	0 (0.0)	1 (1.0)	9 (9.0)	48 (48.0)	42 (42.0)	4.31 ± 0.68	
ChatGPT-generated response in medical education and evidence-based medicine about croup disease							
The validity of this ChatGPT-generated response is high	0 (0.0)	0 (0.0)	11 (11.0)	62 (62.0)	27 (27.0)	4.16 ± 0.6	100 (100%)
The medical education information and the evidence-based medicine (EBM reasoning demonstrated in this ChatGPT-generated response are strong)	0 (0.0)	2 (2.0)	16 (16.0)	53 (53.0)	29 (29.0)	4.09 ± 0.73	
This ChatGPT-generated response is useful for medical education	0 (0.0)	1 (1.0)	12 (12.0)	51 (51.0)	36 (36.0)	4.22 ± 0.69	
This ChatGPT-generated response is useful for trainee’s education	0 (0.0)	2 (2.0)	8 (8.0)	51 (51.0)	39 (39.0)	4.27 ± 0.69	
I believe that pediatric emergency medicine textbooks, such as Fleisher & Ludwig’s Textbook of Pediatric Emergency (8th edition), provide better information	1 (1.0)	8 (8.0)	25 (25.0)	41 (41.0)	25 (25.0)	3.81 ± 0.94	

### Evaluation of ChatGPT’s educational value on croup

Respondents also acknowledged the educational potential of the AI-generated content. Over 90% believed it could benefit trainees and supplement traditional learning. However, 66% still favored textbooks like *Fleisher & Ludwig’s* for deeper understanding of complex cases.

### Comparison between AI users and nonusers

Of the 100 participants, 79.0% reported using AI-based systems, whereas 21.0% had never used such systems (Table 4). The demographic and professional characteristics were compared between the two groups to identify significant differences. In terms of age distribution, 68.4% of AI users were in the 20–39 age group compared with 52.4% of nonusers, whereas 31.6% of AI users were aged ≥ 40 years compared with 47.6% of nonusers. However, the difference was not statistically significant (*p* = 0.173). The sex distribution was similar between the groups, with males comprising 63.3% of AI users and 57.1% of nonusers (*p* = 0.606). Regarding nationality, 81.0% of AI users were Saudi nationals, compared with 61.9% of nonusers, suggesting a trend toward higher AI adoption among Saudi nationals, although this difference did not reach statistical significance (*p* = 0.064).

**Table 4:** Association between sociodemographic characteristics and ever used AI-based systems, such as ChatGPT, in medical practice or education before (*n* = 100).

Characteristic	Description	Never used AI-based systems	Used AI-based systems	*p*-value
		*N* = 21 (21%)	*N* = 79 (79.0%)	
Age (year)	20–39	11 (52.4%)	54 (68.4%)	0.173
	≥ 40	10 (47.6%)	25 (31.6%)	
Sex	Female	9 (42.9%)	29 (36.7%)	0.606
	Male	12 (57.1%)	50 (63.3%)	
Nationality	Non-Saudi	8 (38.1%)	15 (19.0%)	0.064
	Saudi	13 (61.9%)	64 (81.0%)	
Professional level	Assistants/specialists	6 (28.6%)	11 (13.9%)	0.085
	Consultant	11 (52.4%)	34 (43.0%)	
	Fellow	4 (19.0%)	34 (43.0%)	
Educational level	Bachelor’s degree	0 (0.0%)	1 (1.3%)	0.486
	Doctorate or board-certified	7 (33.3%)	39 (49.4%)	
	Fellowship	11 (52.4%)	33 (41.8%)	
	Master’s degree	3 (14.3%)	6 (7.6%)	
Employment	Employed	18 (85.7%)	53 (67.1%)	0.239
	Trainee	3 (14.3%)	25 (31.6%)	
	Unemployed	0 (0.0%)	1 (1.3%)	
Total years of clinical experience in pediatric emergency medicine	Mean ± SD	9.2 ± 5.1	9 ± 4.6	0.844

The professional-level distribution showed some variation between groups, with fellows representing 43.0% of AI users, but only 19.0% of nonusers. Consultants comprised 43.0% of the AI users and 52.4% of the nonusers, whereas assistants/specialists accounted for 13.9% of the AI users and 28.6% of the nonusers. However, these differences were not statistically significant (*p* = 0.085).

Educational background was similar between the groups (*p* = 0.486), with doctorate or board-certified professionals representing 49.4% of AI users and 33.3% of nonusers. Fellowship-trained professionals comprised 41.8% of AI users and 52.4% of nonusers, whereas those with master’s degrees accounted for 7.6% of AI users and 14.3% of nonusers. Employment status showed that trainees represented a higher proportion of AI users (31.6%) than nonusers (14.3%), whereas employed professionals comprised 67.1% of AI users and 85.7% of nonusers. However, this difference was not statistically significant (*p* = 0.239).

The mean years of clinical experience in PEM was nearly identical between AI users (9.0 ± 4.6 years) and nonusers (9.2 ± 5.1 years), with no significant difference (*p* = 0.844).

### Logistic regression analysis for AI system usage

A logistic regression analysis was performed to identify the factors associated with the use of AI-based systems (Table 5). Odds ratios (ORs) with 95% confidence intervals (CIs) were calculated for various demographic and professional characteristics, with no statistical significance. For age, participants aged ≥ 40 years had lower odds of using AI-based systems compared with those aged 20–39 years (OR = 0.74; 95% CI, 0.25–2.22; *p* = 0.589). Males had higher odds of using AI-based systems compared with females (OR = 1.53; 95% CI, 0.53–4.43; *p* = 0.433), whereas Saudi nationals had higher odds compared with non-Saudis (OR = 2.64; 95% CI, 0.45–15.44; *p* = 0.280), although neither reached statistical significance.

**Table 5:** Binary logistic regression analysis predicting the association of ever used AI-based ChatGPT in medical practice or education with the sociodemographic characteristics (*n* = 79).

Characteristic	OR (LL–UL)	*p*-value
Age (year) ≥ 40: 20–39	0.74 (0.25–2.22)	0.589
Sex male: female	1.53 (0.53–4.43)	0.433
Nationality Saudi: Non-Saudi	2.64 (0.45–15.44)	0.280
Professional level Consultant/fellow: assistants/specialists	1.92 (0.29–12.5)	0.495
	2.75 (0.13–60.16)	0.521
Educational level Doctorate or board-certified/fellowship/master’s degree: bachelor’s degree	0 (0–)	1.000
Employment Trainee: employed	1.15 (0.1–13.63)	0.914
Total years of clinical experience in pediatric emergency medicine	1.03 (0.91–1.17)	0.609

Regarding professional level, both consultants (OR = 1.92; 95% CI, 0.29–12.5; *p* = 0.495) and fellows (OR = 2.75; 95% CI, 0.13–60.16; *p* = 0.521) had higher odds of using AI-based systems compared with assistants/specialists, although these differences were not statistically significant. The analysis of educational level did not yield meaningful odds ratios because the reference category (bachelor’s degree) had limited representation in the sample. Similarly, for employment status, trainees had slightly higher odds of using AI-based systems compared with employed professionals (OR = 1.15; 95% CI, 0.1–13.63 L *p* = 0.914), but this was not statistically significant.

Years of clinical experience in PEM showed a minimal association with AI system usage (OR = 1.03; 95% CI, 0.91–1.17; *p* = 0.609), suggesting that experience had little influence on the adoption of AI-based systems.

### Further results on AI usage, concerns, and impact

**Figure 1:**
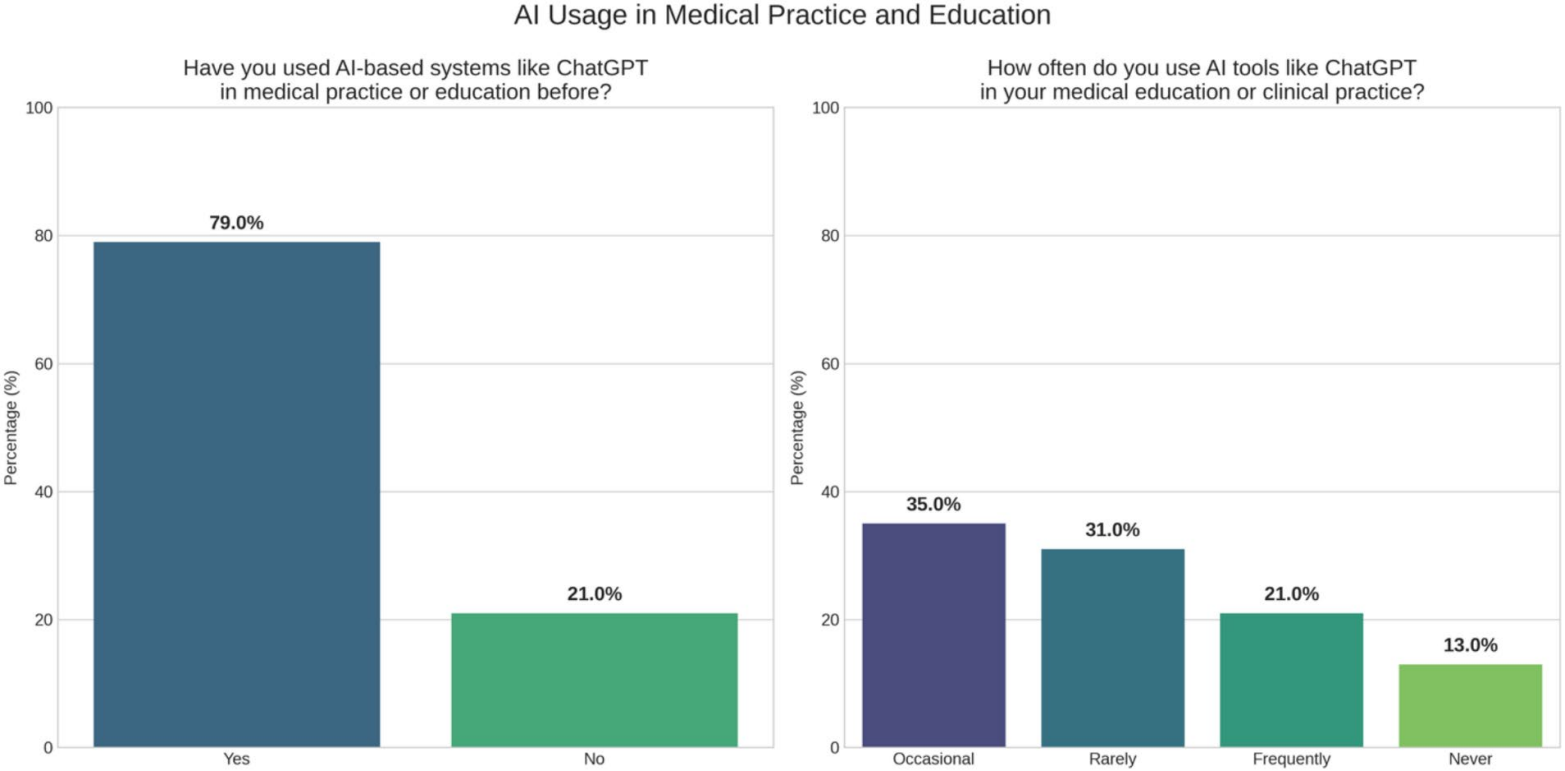
General experience and perception of AI in PEM.

Most participants (79%) reported prior experience using AI-based tools, such as ChatGPT, in medical education or clinical practice. However, frequent use varied considerably; 36% used these tools occasionally, 31% rarely, and 21% frequently, whereas 13% had never used them (Figure 1).

**Figure 2:**
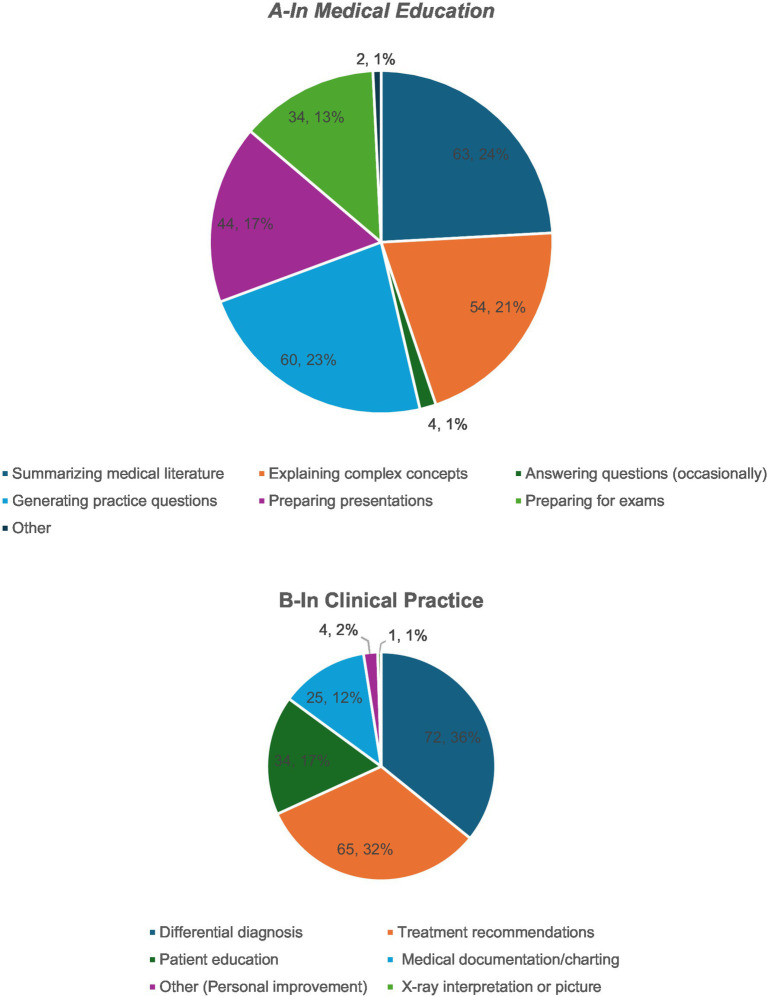
The ways of AI use among PEM physicians in medical education and clinical practice.

In educational settings, AI was primarily used for information retrieval and simplification. The most common use involved answering medical questions (34.3%), followed by summarizing the literature (21.7%), and clarifying complex concepts (14.3%). Additionally, 12% of the respondents employed AI for preparing presentations and another 12% for exam preparation. Less frequent uses included generating practice questions (5.7%) and other individual-specific tasks (2.1%; Figure 2A).

**Figure 3:**
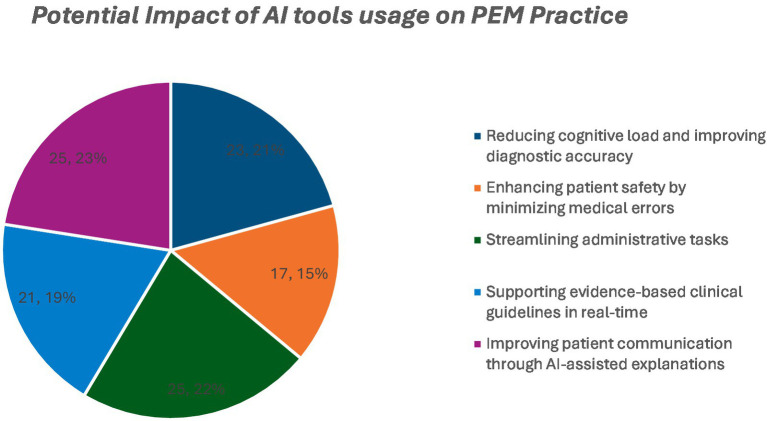
Potential impact of AI tool usage on PEM practice.

Clinical applications of AI reflected its growing implementation into diagnostic and decision-support processes. The most frequently cited use was to develop differential diagnoses (36%), followed by treatment planning (32%), and documentation tasks (17%). AI was also used for patient education (12%) and, to a lesser extent, for professional self-improvement (2%) and image interpretation (1%; Figure 2B). Despite the prevalent optimism toward AI integration, the participants raised concerns chiefly about the accuracy of AI-generated content (83%), patient safety (56%), ethical and legal implications (54%), and reliance on automated systems (45%). Data privacy was cited by 34%, with only 4% reporting no concerns, highlighting the need for caution even among early adopters (Figure 3).

**Figure 4:**
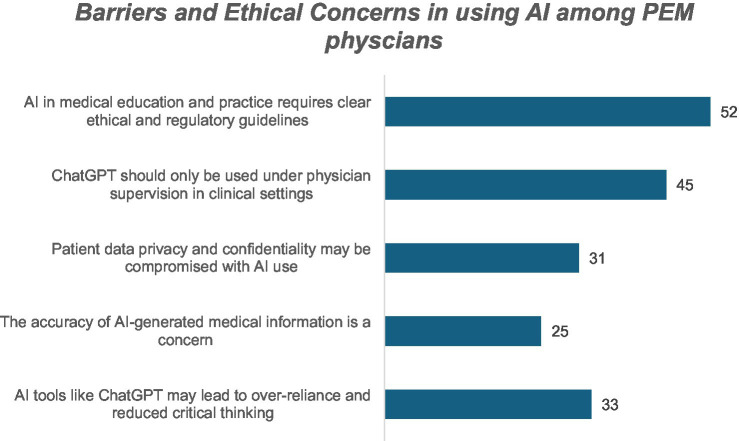
Barriers and ethical concerns in using AI among PEM physicians.

The participants anticipated AI’s benefits in reducing cognitive workload (23%), improving diagnostic accuracy (22%), promoting guideline adherence (21%), reducing errors (21%), enhancing patient communication (19%), and saving administrative time (15%; Figure 4).

Despite these advantages, key structural and ethical barriers were identified. The most frequently reported was the lack of formal regulatory or ethical guidance (52%). Many participants emphasized the importance of physician oversight in AI-supported care (45%). Concerns regarding patient data protection (31%) and the accuracy of AI-generated outputs (25%) persisted, and 33% expressed apprehension that AI could eventually diminish clinicians’ critical thinking skills. These findings underscore the importance of pairing AI integration with governance frameworks, education, and clinical safeguards to ensure its responsible use.

## Discussion

This study explored the perceptions of pediatric emergency physicians in Saudi Arabia toward the use of AI, specifically ChatGPT, in clinical practice and medical education. The findings revealed a highly positive outlook, with 96% of respondents agreeing that AI will play a major role in the future of PEM. This aligns with global trends showing the growing acceptance of AI tools among healthcare professionals, particularly in high-pressure specialties, such as emergency medicine, where rapid decision-making is crucial (6).

In medical education, the participants rated ChatGPT highly because of its ability to provide rapid access to information, support critical thinking, and simulate clinical cases. More than 90% believed that learning could be enhanced by supplementing traditional methods. These findings are consistent with the literature showing that AI-based platforms, when integrated thoughtfully, can improve engagement, personalize learning, and bridge knowledge gaps (3, 4). However, approximately two-thirds of the respondents still preferred using traditional textbooks in complex scenarios, suggesting that although ChatGPT is a useful adjunct, it has not yet been seen as a replacement for established academic resources (5).

Recent studies have supported similar insights. Abdelhafiz et al. (9) found that medical students across disciplines showed strong interest in ChatGPT’s academic potential. Kıyak and Emekli (10) reviewed the validity of ChatGPT prompts in generating multiple-choice questions and educational scenarios. Additionally, Gibson et al. (11) evaluated ChatGPT’s usefulness in creating patient education materials, demonstrating its broad applicability across medical fields. These findings reinforce the utility of LLMs while underscoring the need for proper validation frameworks.

In clinical practice, AI has been well-received for its potential in differential diagnosis, documentation, and decision support. Over 77% of the physicians agreed that AI could assist with diagnosis and treatment planning in PEM. These perceptions reflect international studies demonstrating AI’s ability to improve diagnostic accuracy and efficiency while reducing the administrative burden (12, 13). Additionally, 78% of the participants found AI useful in documentation tasks, echoing studies showing that AI integration can streamline clinical workflows and free up more time for patient care (13, 14).

Notably, despite favorable ratings of the ChatGPT-generated response to croup management, 67% of the participants believed that they could provide better clinical answers. This finding reflects cautious optimism; physicians recognize the value of AI but still trust their clinical expertise over automated recommendations. This aligns with the broader concerns in the literature regarding the risk of overreliance on AI tools and the need for continuous human oversight (15).

The participants also voiced significant concerns about the use of AI, particularly regarding accuracy (83%), patient safety (56%), ethical implications (54%), and data privacy (34%). These concerns are echoed in global discussions on AI regulation, emphasizing the need for validated, transparent algorithms and clearly defined clinical governance frameworks (16, 17). In the Saudi context, the lack of local regulatory guidelines and Arabic-language interfaces has been identified as a key barrier to its broader adoption. As the Kingdom continues to advance its Vision 2030 digital health goals, addressing these gaps is essential for safe and culturally competent AI integration (8, 18).

Overall, the high acceptance of AI among Saudi PEM physicians, coupled with the identified concerns, indicates a readiness for adoption paired with a demand for ethical and operational safeguards. To ensure a responsible implementation, future efforts should focus on establishing national standards, incorporating AI literacy into medical curricula, and continuously evaluating AI tools in real-world clinical settings.

### Limitations

This study has some limitations. First, the cross-sectional design restricts causal inference. Second, the study focused solely on pediatric emergency physicians in Saudi Arabia, limiting broader generalizability. Furthermore, by focusing solely on PEM physicians, the study does not capture AI perceptions in other critical emergency disciplines such as adult emergency medicine, cardiology, or neurology. Future research should expand into these areas for a more comprehensive understanding of AI adoption in emergency care. Third, a single standardized prompt and a fictional scenario were used to assess ChatGPT’s utility, which may not capture the full variability of LLM responses. The AI response was generated at a specific time (April 2024) using ChatGPT-4o, and future outputs may differ due to rapid LLM evolution. Lastly, as the study relied on self-reported perceptions, response bias may have influenced participant assessments. Although the sample size met the calculated minimum for statistical validity, a larger sample would allow for more robust subgroup analysis and improve generalizability.

## Conclusion

PEM physicians in Saudi Arabia are significantly positive about integrating ChatGPT in clinical and educational settings. Their perception suggests that ChatGPT is not only an efficient information tool but also a valuable aid for training and patient care. Although trust in AI is conditional and concerns about regulation, accuracy, and ethics persist, the willingness to adopt such technologies reflects a shift in clinical culture. Policymakers, educators, and healthcare leaders should capitalize on this momentum to establish a supportive infrastructure, training modules, and ethical frameworks to guide the safe and effective use of AI in health care. As AI evolves, continued evaluation and adaptation are necessary to ensure that ChatGPT contributes to better patient outcomes and clinician support.

## Data Availability

The raw data supporting the conclusions of this article will be made available by the authors, without undue reservation.
