# Supplementary material for: Perceptions of large language models in medical education and clinical practice among pediatric emergency physicians in Saudi Arabia: a multiregional cross-sectional study
Source: Front Public Health. 2025 Jul 30;13:1634638. doi: 10.3389/fpubh.2025.1634638 (PMC12343484; doi:10.3389/fpubh.2025.1634638)
Supplement: Supplementary file 1 [file Table_1.DOCX]

Supplementary Appendix A

## **Section 1: Demographics**

**1. Age:** [*](https://www.google.com/url?sa=t&source=web&rct=j&opi=89978449&url=https://www.sciencedirect.com/topics/computer-science/chronological-age&ved=2ahUKEwiHpaXLo82LAxXRSfEDHdcMLE4QFnoECBwQAw&usg=AOvVaw1oXGOckvbZCAyv2kE9yfJp)

 Early Adulthood (20–39)

 Middle Adulthood (40–59)

 Older adults (60+ years)

**2. Gender:**

 Male

 Female

**3. Nationality:**

 Saudi

 Non-Saudi

**4. Marital status:**

 Single

 Married

 Widowed

 Separated

 Divorced

**5.What is your current professional status?**

 Fellow

 Assistant/specialist

 Consultant

 Others (please specify): ______

**6. Highest level of education:**

 Diploma

 Bachelor’s degree

 Master’s degree

 Doctorate or board-certified

 Fellowship

**7. Which region you are from?**

 Central region

 Western region

 Eastern region

 Northern region

 Southern region

**8.Employment status:**

 Trainee

 Unemployed

 Employed

 Retired

**9. Total years of clinical experience in pediatric emergency medicine. __________years**

**10. Have you used AI-based systems like ChatGPT in medical practice or education before?**

 Yes

 No

**11. How often do you use AI tools like ChatGPT in your medical education or clinical practice?**

 Never

 Rarely

 Occasionally

 Frequently

**12. I believe AI tools like ChatGPT will play a major role in the future of PEM.**
 Yes

 No

**13. In what ways do you use AI-generated responses like ChatGPT for medical education? (***Select all that apply***)**

 Summarizing medical literature
 Generating practice questions
 Explaining complex concepts
 Preparation for examinations

 Preparing for presentations
 Other (please specify): ________

**14. In which clinical areas have you used AI tools like ChatGPT? (Select all that apply)**

 Differential diagnosis
 Treatment recommendations
 Patient education
 Medical documentation/charting
 Other (please specify): ________

**15. What are your main concerns about using ChatGPT in clinical practice? (***Select all that apply***)**

 Accuracy of information
 Ethical and legal issues
 Patient safety risks
 Privacy and confidentiality
 Over-reliance on AI
 Other (please specify): ________

## **Section 2: AI Use in Medical Education and Clinical Practice**

**16. Please indicate your level of agreement with the following statements regarding AI in medical education:**

- AI tools like ChatGPT can enhance medical education by providing quick access to medical knowledge.
- AI tools like ChatGPT improve decision-making and critical thinking skills in trainees.
- AI tools like ChatGPT can be used effectively to simulate patient cases for clinical learning.
- AI tools like ChatGPT can supplement traditional teaching methods in PEM training.
- AI tools like ChatGPT helps in staying updated with the latest medical guidelines and literature.
- I believe AI-powered tools like ChatGPT should be integrated into medical education curricula.

Response Options:

 Strongly Agree

 Agree

 Neutral

 Disagree

 Strongly Disagree

**17. Please indicate your level of agreement with the following statements regarding AI in medical practice:**

- ChatGPT medical responses are generally accurate.
- I am comfortable using AI-based tools like ChatGPT for clinical decision support.
- AI like ChatGPT can assist in differential diagnosis and treatment recommendations in PEM.
- AI like ChatGPT can improve efficiency in documentation and chart reviews.
- AI-driven medical chatbots should be integrated into PEM workflows.
- I would trust AI-generated recommendations if validated by evidence-based medicine.

Response Options:

 Strongly Agree

 Agree

 Neutral

 Disagree

 Strongly Disagree

## **Section 3: Perception of AI-Generated Clinical Response**

**18. Below is a ChatGPT-generated response to the following clinical question:**

Prompt: “***Please act as a doctor and provide general knowledge, natural history, and a detailed treatment plan for a 3-year-old boy with croup***.”

**[ChatGPT’s Response]**

### **General Knowledge on Croup**

- Croup is a common viral respiratory illness that primarily affects young children, typically between 6 months and 3 years of age. It is characterized by inflammation and swelling of the upper airways, particularly the larynx, trachea, and bronchi. This inflammation leads to **barking cough, stridor (a high-pitched sound during inspiration), hoarseness,** and, in severe cases, **respiratory distress.**

**Etiology (Causes)**

- **Most common cause: Parainfluenza virus types 1 and 2** (responsible for majority of cases)
- Other viral causes: Respiratory syncytial viruses (RSV), adenoviruses, influenza viruses, rhinoviruses, enteroviruses, and coronaviruses.
- Croup is most prevalent during the **fall and winter seasons** and spreads through respiratory droplets.

**Natural History of Croup**

- Croup is generally a **self-limiting** illness with a **gradual onset over 12–48 h,** following a predictable course:

**1.Early Symptoms (1–2 days before stridor develops)**

- Mild fever
- Runny nose
- Mild cough

**2.Progressive Symptoms (Day 2–3)**

- **Barking cough** (worsening at night)
- Hoarseness
- Inspiratory **stridor** (mild-to-severe)
- Increased work of breathing in moderate to severe cases (e.g., nasal flaring and intercostal retraction)

**3.Resolution (3–7 days)**

- Symptoms peak within **48 h** and gradually improve.
- Cough and hoarseness may persist for **up to 1 week.**

**Severity Classification of Croup**

- Croup is classified based on symptoms and severity:

| Severity | Barking Cough | Stridor | Work of Breathing | Mental Status | Oxygen Saturation |
| --- | --- | --- | --- | --- | --- |
| **Mild** | Present | None or only when agitated | None or minimal | Normal | >92% |
| **Moderate** | Frequent | At rest | Mild to moderate retractions | Normal | >92% |
| **Severe** | Frequent | At rest and loud | Marked retractions, tachypnea | Agitated or lethargic | <92% |

### **Treatment Plan for a 3-Year-Old Boy with Croup**

### Management depends on the severity. Most cases are **mild and can be managed at home**, whereas **moderate to severe cases require hospitalization**.

### 1. ***Mild Croup* (No Stridor at Rest) → Home Management**

### **1. Supportive Care**

### Keeping the child **calm** (crying worsens symptoms).

### **Cool mist therapy** or **humidified air** may soothe the airways.

### Ensure adequate **hydration** (fluids, breast milk, or formula, if appropriate).

### **Antipyretics** for fever (e.g., **Acetaminophen** or **Ibuprofen**).

### **2. Medications (If Needed)**

### Single dose of **Dexamethasone** (0.15–0.6 mg/kg PO, max 10 mg) helps reduce inflammation.

### 3. **When to Seek Medical Care?**

### Persistent stridor

### Increased breathing effort

### Poor oral intake

### Lethargy and cyanosis.

### *2.* ***Moderate Croup* (Stridor at Rest, Mild Retractions) → Emergency Department (ED) Management**

### **Dexamethasone (PO/IM):**

### **Dose:** 0.6 mg/kg (max 10 mg)

### Takes 4–6 h for full effect.

### **Nebulized Epinephrine (for stridor at rest):**

### **L-Epinephrine 1:1000 (5 mL via nebulizer)**

### Reduces airway swelling rapidly.

### **Requires observation** at least 3–4 h of monitoring post-administration.

### **Oxygen Therapy: Only if hypoxic (SpO_2_ <92%)**: humidified oxygen via facemask.

### *3.* ***Severe Croup* (Stridor at Rest + Severe Retractions or Cyanosis) → Urgent Hospital Management**

### **Nebulized Epinephrine (as mentioned above, may require repeated doses).**

### **Dexamethasone (IM/IV) if the child cannot tolerate oral dosing**

### **Close monitoring for signs of respiratory failure**

### Consider ICU admission if the child’s condition deteriorates.

### Intubation is **rarely required** but may be necessary in cases of impending airway obstruction.

**Please indicate your level of agreement with the following statements regarding the ChatGPT-generated response:**

The validity of this ChatGPT-generated response is high.

The clinical reasoning demonstrated in this ChatGPT-generated response is strong.

This ChatGPT-generated response is useful for clinical application.

This ChatGPT-generated response is useful for patient education.

I believe I can provide a better response than the ChatGPT-generated response for a given clinical scenario.

This AI-generated response from ChatGPT could be beneficial for medical education.

(Responses: )

[ ] Strongly Agree

 Agree

 Neutral

 Disagree

 Strongly Disagree).

**19. Below is a ChatGPT-generated response to the medical education question:**

## Prompt: “*Please provide a updated summary of the latest literature and evidence-based guidelines on croup management in children, including references*”

**[ChatGPT’s Response]**

Recent literature provides the following evidence-based recommendations for croup management:

**Corticosteroids:** Administer a single dose of dexamethasone (0.15 mg/kg to 0.6 mg/kg) orally to all children diagnosed with croup, regardless of severity. This approach reduces the symptom duration and decreases the need for additional interventions.^1^

**Nebulized Epinephrine:** Effectively reduces airway obstruction in moderate-to-severe croup Benefits are typically observed within 30 min, although the effects may diminish after 2 h. Therefore, continuous monitoring is essential.^1^

**Supportive Care:** Maintaining a calm environment is crucial, as agitation can exacerbate symptoms. While humidified air has traditionally been used, current evidence does not support its routine use.^2^

**Avoidance of Unnecessary Interventions:** Routine use of antibiotics, antitussives, decongestants, and imaging studies is discouraged unless there is a clear indication of bacterial infection or an alternative diagnosis.^1^

Implementing these practices can enhance patient outcomes and reduce unnecessary treatments in croup management.

## *The Pharmaceutical Journal, PJ August 2024, 313 (7988), 313(7988)::DOI:10.1211/PJ.2024.1.325623*

## *Ortiz-Alvarez, O. Acute Management of Croup in the Emergency Department. Paediatr. Child Health June 1 2017, 22 (3), 166–173. DOI: 10.1093/pch/pxx019*

**Please indicate your level of agreement with the following statements regarding ChatGPT-generated response:**

The validity of this ChatGPT-generated response is high.

1. the medical education information and the evidence-based medicine (EBM) reasoning demonstrated in this ChatGPT-generated response is strong.

This ChatGPT-generated response is useful for medical education.

This ChatGPT-generated response is useful for trainee’s education.

1. I believe that pediatric emergency medicine textbooks, such as **Fleisher & Ludwig's Textbook of Pediatric Emergency (8^th^ edition)** provide better information than the ChatGPT-generated response for the given disease.

Responses:

 Strongly Agree

 Agree

 Neutral

 Disagree

 Strongly Disagree.

## Potential Impact of AI tools on PEM Practice

**20. To what extent do you believe AI will impact the following areas?**

- Reducing cognitive load and improving diagnostic accuracy.
- Enhancing patient safety by minimizing medical errors.
- Streamlining administrative tasks (e.g., documentation).
- Supporting evidence-based clinical guidelines in real-time.
- Improving patient communication through AI-assisted explanations.

Responses:

 Strongly Agree

 Agree

 Neutral

 Disagree

 Strongly Disagree

## **Barriers and Ethical Concerns**

**21. How much do you agree with the following concerns?**

- AI tools like ChatGPT may lead to over-reliance and reduced critical thinking.
- The accuracy of AI-generated medical information is a concern.
- Patient data privacy and confidentiality may be compromised with AI use.
- ChatGPT should only be used under physician supervision in clinical settings.
- AI in medical education and practice requires clear ethical and regulatory guidelines.

Responses:

 Strongly Agree

 Agree

 Neutral

 Disagree

 Strongly Disagree

## **Section 4: Open-Ended Questions**

**22. Do you have any additional comments regarding the use of AI, such as ChatGPT, in medical education and clinical practice?**
